# Approach to impaired corollary discharge in patients with schizophrenia: An analysis of self-induced somatosensory evoked potentials and fields

**DOI:** 10.3389/fpsyg.2022.904995

**Published:** 2022-08-17

**Authors:** Kazuyori Yagyu, Atsuhito Toyomaki, Naoki Hashimoto, Hideaki Shiraishi, Ichiro Kusumi, Harumitsu Murohashi

**Affiliations:** ^1^Department of Pediatrics, Hokkaido University Hospital, Sapporo, Hokkaidō, Japan; ^2^Department of Child and Adolescent Psychiatry, Hokkaido University Hospital, Sapporo, Hokkaidō, Japan; ^3^Department of Psychiatry, Hokkaido University, Graduate School of Medicine, Sapporo, Hokkaidō, Japan; ^4^Department of Human Development Sciences, Hokkaido University, Graduate School of Education, Sapporo, Hokkaidō, Japan

**Keywords:** schizophrenia, somatosensory evoked potential, somatosensory evoked field, centrencephalic system, corollary discharge, primary sensorimotor area

## Abstract

**Background:**

Difficulty in distinguishing between self-generated actions and those generated by others is a core feature of schizophrenia. This is thought to be underpinned by the failure of corollary discharge. However, few studies have investigated these events using somatosensory evoked potentials (SEPs) and somatosensory evoked magnetic fields (SEFs).

**Methods:**

The study included 15 right-handed patients with schizophrenia and 16 healthy controls. SEP and SEF were elicited by electrical stimuli to the left median nerve at intervals of 1–3 s. In the external condition, stimuli were externally induced by a machine. In the self-condition, stimuli were induced by tapping the participants’ own right index finger. Peak amplitude at C4’ in SEP and root mean square in 10 channels on the right primary somatosensory area in SEF were analyzed.

**Results:**

Although there was a significant main effect of condition at N20m, and a significant main effect of condition and group at P30m, no significant interactions of condition and group were found in either N20m or P30m. The *post-hoc* Wilcoxon signed-rank test revealed that the peak value of P30m in the external condition was significantly higher than that in the self-condition in the healthy control group only. In addition, there was a significant positive correlation between the peak value of P30m in the self-condition and a positive symptom score.

**Conclusion:**

In the current study, we did not find abnormalities of corollary discharge in primary sensory areas in patients with schizophrenia. Further investigations with more cases may reveal the possibility of corollary discharge disturbance in the primary sensory cortex.

## Introduction

It is essential to be able to distinguish self-derived stimuli from stimuli from the environment (Crapse and Sommer, 2008). This differentiation is thought to be achieved by the generation of blueprints of motor commands in the brain, known as efference copies, which are transmitted to sensory reception structures. Expected sensations resulting from movements are then effectively subtracted from the real sensations, resulting in net suppression of the sensation arising from one’s own actions, which is known as corollary discharge (Ford et al., 2014; Parlikar et al., 2019). Corollary discharge is not directly observed in humans; however, its function is demonstrated as attenuated electrophysiological response, known as gating effect (Straka, 2018). This neural integration feed-forward mechanism was initially extensively studied in the field of visual perception (Sperry, 1950) and later spread to other sensory areas such as auditory perception and proprioception (Wolpert and Miall, 1996; Numminen et al., 1999). The ability to establish the concept of self and non-self is also thought to incorporate complex higher mental functions like thinking and consciousness that, in principle, retain the features central to motor mechanisms (Parlikar et al., 2019). Additionally, electroencephalography (EEG) and magnetoencephalography (MEG) have been used in human electrophysiological studies to observe neural activity ~100 ms preceding a motor act; this can help elucidate the innate physiological state of efference copies and corollary discharge.

Schizophrenia is a complex behavioral and cognitive syndrome that is known to affect approximately 0.3 to 0.7% of the population worldwide (Owen et al., 2016). Difficulty in distinguishing between thoughts and actions generated by oneself and others is one of the core features of schizophrenia (Taylor, 2011). It is thought to be underpinned by the failure of corollary discharge (Feinberg, 1978; Frith, 1995), and accompanied with positive symptoms such as hallucinations (Ford, 2016) and delusions (Farrer et al., 2004), and negative symptoms such as avolition and apathy (Ford et al., 2008).

Most previous studies have focused on the role of corollary discharge dysfunction in the pathophysiology of auditory hallucinations in schizophrenia. In healthy controls, activity levels of N1 in EEG-based event-related potentials and M100 in MEG-based responses are lower while speaking than when recorded sounds are played back to the speaker (Curio et al., 2000; Heinks-Maldonado et al., 2005). Ford and colleagues showed that N1 suppression while talking is reduced in patients with schizophrenia, which means that the efference copy may not function correctly (Ford et al., 2001a,b, 2007; Ford and Mathalon, 2004). They also observed N1 suppression in healthy controls by comparing N1 to tones delivered by button presses with N1 to these tones played back, but this was not observed in patients with schizophrenia (Ford et al., 2014).

Other studies have investigated the relationship between corollary discharge dysfunction and passivity symptom in patients with schizophrenia. Passivity symptom refers to an impaired sense of agency (Farrer et al., 2004), which is one of the social cognitive domains that are impaired in schizophrenia (Green et al., 2008, 2015). Patients with schizophrenia tend to misattribute the agency of their own volitional act to an external force. In one study, healthy subjects and psychiatric patients with neither auditory hallucinations nor passivity experienced self-produced stimuli as less intense than stimuli by another person, whereas patients with these symptoms did not report such a difference (Blakemore et al., 2000). Other experimental tasks have evaluated the explicit experience of a sense of agency when there is a discrepancy in time or angle between an intended action and its effect on a screen (Farrer et al., 2004; Koreki et al., 2015). These experimental paradigms showed that patients with schizophrenia were more likely than healthy individuals to believe that the effect on the screen was self-derived, even when time or angle deviations were greater. An fMRI study that examined the differences in brain activation when sensation and action were synchronous (self-condition) compared with when the former occurred after an unexpected delay (non-self-condition) showed attenuated activation in the secondary somatosensory cortex in healthy controls but not in patients with schizophrenia (Shergill et al., 2014). These findings suggest abnormalities in sense of agency, arising from the somatosensory system, in patients with schizophrenia. Ford and colleagues reported that using button pressing task for schizophrenia and control group, prepress gamma-band neural synchrony was (1) maximal over the contralateral sensory-motor cortex in healthy subjects, (2) correlated with the ipsilateralized somatosensory ERP amplitude evoked by the press, and (3) reduced in patients (Ford et al., 2008). These results show a possible deficit of corollary discharge around the primary sensorimotor area in schizophrenia group. However, their results were obtained by EEG and post-stimulus changes were demonstrated in wide-range periods (75-150 ms). Therefore, it is unclear whether it is a primary somatosensory gate deficit or otherwise.

In the present study, we examined differences in somatosensory evoked fields (SEFs) and somatosensory evoked potentials (SEPs) in the primary somatosensory area elicited by self-derived stimuli and external stimuli in healthy controls and patients with schizophrenia. We predicted that the peak value of SEF and amplitude of SEP in response to self-derived stimuli would be reduced compared to those derived from external stimuli in healthy controls but not in patients with schizophrenia; this lack of reduction may be associated with positive psychotic symptoms in patients with schizophrenia.

## Materials and methods

The Hokkaido University Hospital Ethical Committee approved all study procedures, and the study was conducted according to the principles expressed in the Declaration of Helsinki. A description of the study was given to all participants prior to the study, and written informed consent was obtained.

### Participants

Fifteen patients (four women) who met the criteria for a diagnosis of schizophrenia according to the Diagnostic and Statistical Manual of Mental Disorders, Fourth Edition, Text Revised (DSM-IV-TR) were recruited from Hokkaido University Hospital between January 2010 and March 2011. Patients who had a history of head injury, seizure disorder, dementia, diabetes mellitus, or other significant laboratory results were excluded from the study. No patients had a history of substance misuse, including alcohol abuse. Patients who were currently suicidal and women who were pregnant or breastfeeding were also excluded. All patients were taking second-generation antipsychotics.

For the control group, sixteen patients (five women) with no history of a DSM-IV-TR Axis I disorder were recruited from the community. None of the healthy controls had a neurological disorder, and were not receiving psychotropic medications.

The demographic data of the participants are shown in Table 1. There were no statistical differences in age and sex between the two groups, although the estimated IQ of the control group was slightly higher than the patient group.

**Table 1:** Demographic background of healthy controls and patients with schizophrenia.

	Healthy control	Schizophrenia	Values of *p*
*N* (Women)	*N* = 16 (Women = 5)	*N* = 15 (Women = 4)	*P* = 0.7964^3^
Age (years)	30.5 (5)	31.2 (6.6)	*P* = 0.78^4^
Estimated IQ^1^	113.1 (7.8)	105.8 (9.3)	*P* = 0.04^4^
Duration of disease (years)	NA	9.9 (6.7)	NA
Antipsychotics (mg/day)^2^	NA	742.8 (513.1)	NA
PANSS			
Total	NA	72.3 (12.8)	NA
positive symptoms score	NA	15.9 (3.7)	NA
negative symptoms score	NA	18.3 (4.2)	NA
general psychopathology score	NA	38.1 (6.6)	NA

Values are given as mean (S.D). PANSS: Positive and Negative Syndrome Scale.

1 IQ estimated from the Japanese Adult Reading Test (JART);

2 Chlorpromazine equivalent daily dose;

3 *χ*^2^test;

4 Wilcoxon rank-sum test.

### MEG and EEG data acquisition

MEG data were recorded using a 204-channel, helmet-shaped gradiometer (Vectorview System, Elekta-Neuromag Oy, Stockholm, Sweden) while patients were in a supine position in a magnetically shielded room. During the MEG recording, scalp EEGs were also recorded simultaneously at F3, F4, C3, C4, C3′, C4′, and Cz. The C3′ and C4′ electrodes were placed at 2 cm posterior to C3 and C4 over the somatosensory cortices. MEG and EEG were recorded with a 1,000 Hz sampling rate.

### SEP and SEF recordings

Painless electrical stimuli were delivered to the left median nerves via electrode pairs taped to the left wrist, separated by 2.3 cm. The stimuli were square-wave pulses (0.20 ms) generated by a bipolar stimulator SEM-4201 (NIHONKOHDEN Co. Ltd.). The intensity of the stimulation was adjusted as mild thumb twitches were obtained.

We had two experimental conditions - the external condition and self-condition. In both conditions, stimulations were delivered over 300 times. In the external condition, electrical stimulation was evoked at randomized irregular intervals (1–3 s) induced by a computer (UNIX machine). In the self-condition, electrical stimulation was evoked by tapping the right forefinger when sensed by an infrared sensor. Participants performed right forefinger tapping at a 1–3 s interval, according to their own timing, in the presence of an examiner. Preceding the recording, participants practiced this procedure several times. A trigger from the stimulator was sent to the data acquisition system for signal averaging.

### Data analysis

**Figure 1:**
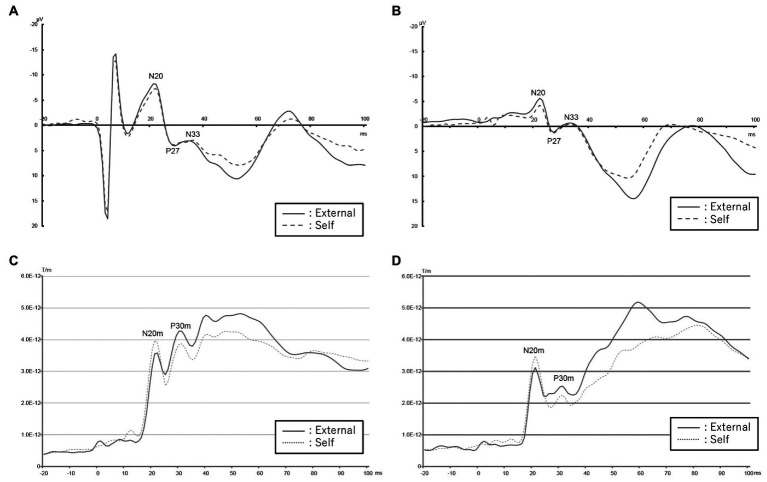
EEG and MEG data acquired from patients with schizophrenia compared to healthy controls in the self and external conditions. **(A)** Mean EEG waveform in healthy controls. Red colored solid line shows external condition and blue colored dashed one shows self-condition. **(B)** Mean EEG waveform in patients with schizophrenia. Red colored solid line shows external condition and blue colored dashed one shows self-condition. **(C)** Root mean square of SEF values of control group. Blue line indicates external condition and red indicates self-condition. **(D)** Root mean square of SEF values of schizophrenia group. Blue line indicates external condition and red indicates self-condition.

EEG and MEG were analyzed separately. Acquired EEG data were analyzed with BESA 5.2.^1^ After filtering frequencies of 10–100 Hz and rejecting artifacts of over 500 μv, offline averaging was done at the C4’ electrode with a baseline of −100 – −10 ms. To remove motion artifacts while tapping, high-cut filtering was set at 10 Hz. After averaging, peak analyses and peak-to-peak analyses were conducted. These filtering settings and analysis methods were to avoid artifacts of right-hand motion and electrical stimulation (Figures 1A,B). MEG data were analyzed using Graph (Elekta-Neuromag Oy, Stockholm, Sweden). After filtering frequencies of 1.5–150 Hz and rejection of artifacts over 10,000 fT/cm, the averaged data with baseline-100–0 ms were presented with X-plots (Elekta-Neuromag Oy, Stockholm, Sweden). While conducting MEG, a pair of gradiometer sensors were set perpendicular to each other. Thus, 5 pairs (i.e., 10 sensors) with a significant N20m component of 204 channels were selected. From these selected data, the root mean square was calculated. We used root mean square because each angle between the MEG sensor and the direction of the local source was diverse, and if the direction of the sensors were opposite, it would have brought about opposite vectors.

### Statistical methods

The background and baseline data were compared between patients with schizophrenia and healthy controls, using the χ^2^ test for categorical variables, and the Wilcoxon rank-sum test for continuous variables. The MEG [peak values of SEF (N20m and P30m)] and EEG data amplitudes of SEP (N20, P27, and N33) were subjected to a two-way ANOVA with condition (self vs. external) as the within-participant factor, and group (schizophrenia vs. healthy control) as the between-participant factor. Additionally, since 8 out of 16 group values did not follow the normal distribution (Supplementary Table 1), we conducted a nonparametric comparison using the Wilcoxon signed-rank test to compare the differences of amplitude according to condition in each group. For all analyses, the level of statistical significance was set to *p* = 0.05. All analyses were performed with R (version 3.6.1).^2^

### Positive and negative syndrome scale and the Japanese version of national adult reading test

To investigate the symptoms of patients with schizophrenia in detail, PANSS scores were acquired by certificated psychiatrists (Hashimoto et al., 2020). To estimate the IQ of healthy controls and the premorbid IQ of patients with schizophrenia, JART scores (Matsuoka et al., 2006) were acquired by skilled testers. Spearman’s rank correlations were used to analyze the relationship between PANSS score and the peak value of SEF or amplitude of SEP, and the relationship between demographic variables (age, estimated IQ, duration of disease, and chlorpromazine equivalent daily dose of antipsychotic).

## Results

### Somatosensory evoked field

N20m and P30m components were observed in almost all participants except for a control participant with an obscure P30m (Figures 1C, D).

#### N20m

In the ANOVA analysis, the main effect of condition [*F*(1,58) = 7.88; *p* = 0.009] was significant; however, the main effect of group [*F*(1,58) = 0.24; *p* = 0.625] and the interaction of condition and group [*F*(1,8) = 0.21; *p* = 0.648] were not significant. There was no significant difference between the peak values of SEF in the self and external conditions in the Wilcoxon signed-rank test in the healthy control group and in the schizophrenia patient group (Table 2).

**Table 2:** Results of ANOVA Wilcoxon signed-rank test for N20 and P30 (external vs. self-conditions).

	Healthy controls		Schizophrenia		ANOVA									*Post-hoc* Wilcoxon signed-rank test (external vs. self)^1^	
					Condition			Group			Condition x Group				
	External	Self	External	Self	*F*	df	*p*	*F*	df	*p*	*F*	df	*p*	Healthy controls	Schizophrenia
SEF															
N20m	3.86 (2.24)	4.38 (1.43)	3.61 (0.97)	3.98 (1.43)	7.88	1,58	0.009	0.24	1,58	0.625	0.21	1,58	0.65	0.058	0.188
P30m	5.31 (2.55)	4.73 (2.01)	3.05 (2.2)	2.71 (2.01)	4.79	1,56	0.037	5.98	1,56	0.021	0.32	1,56	0.58	0.022	0.296
SEP															
N20	−6.91 (4.31)	−5.36 (4.91)	−5.91 (5)	−4.98 (3.87)	8.68	1,58	0.006	0.18	1,58	0.676	0.55	1,58	0.47	0.013	0.064
P27	3.61 (2.51)	4.21 (3.09)	1.71 (2.67)	2.02 (2.05)	1.63	1,58	0.212	5.14	1,58	0.031	0.15	1,58	0.70	0.105	0.489
N33	−1.52 (2.91)	−2.16 (3.47)	−3.3 (3.01)	−3.25 (2.62)	0.56	1,58	0.459	1.88	1,58	0.181	0.69	1,58	0.41	0.323	0.89

Values are given as mean (S.D). ANOVA: Analysis for variance; SEF: somatosensory evoked field; SEP: somatosensory evoked potential.

1 value of *p* for Wilcoxon signed-rank test.

#### P30m

In the ANOVA analysis, the main effect of condition [*F*(1,56) = 4.79; *p* = 0.037] and the main effect of group [*F*(1,56) = 5.98; *p* = 0.021] were significant, though the interaction of condition and group [*F*(1,56) = 0.32; *p* = 0.576] was not significant. The Wilcoxon signed-rank test revealed that the peak value of P30m in the external condition was significantly higher than that in the self-condition in the healthy control group (*p* = 0.022); however, there was no significant difference in the schizophrenia group (*p* = 0.296; Table 2).

#### Correlations between PANSS and SEF

**Figure 2:**
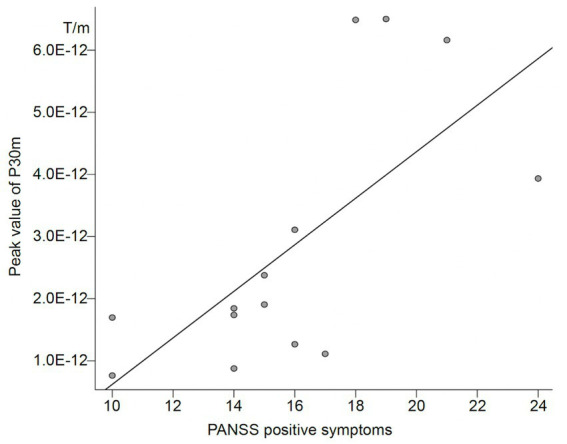
Correlation between PANSS positive symptom score and peak value of P30m in patients with schizophrenia.

In the schizophrenia group, the correlation between PANSS sub scores and peak values of P30m and N20m in the external and self-conditions was examined. We found a positive correlation between positive symptom score and P30m component in the self-condition (Spearman’s rho = 0.72; *p* = 0.004; Table 3; Figure 2). There was no correlation between demographic and peak value of P30m or N20m.

**Table 3:** Spearman’s correlation coefficient for SEF (N20m, P30m) amplitude and demographic factors.

	N20m		P30m	
	External	Self	External	Self
Age (years)	0.16	0.24	0.06	0.12
Estimated IQ^1^	0.19	0.36	0.21	0.36
Duration of disease(years)	0.24	0.46	0.5	0.16
Antipsychotics (mg/day)^2^	0.06	0.23	0.24	0.36
PANSS				
Total	−0.24	0.38	0.21	0.35
positive symptoms score	0.29	0.14	0.3	0.72^3^
negative symptoms score	0.22	−0.48	0.04	0.12
general psychopathology score	−0.26	0.31	0.04	0.24

PANSS: Positive and negative syndrome scale.

1 IQ estimated from the Japanese Adult Reading Test (JART);

2 Chlorpromazine equivalent daily dose;

3 *p* < 0.005.

### Somatosensory evoked potential

We checked each averaged waveform and detected N20, P27, and N33. In the ANOVA analysis, there was a significant main effect of condition [*F*(1,58) = 8.68; *p* = 0.006] in N20, and a significant main effect of group [*F*(1,58) = 5.14; *p* = 0.031] in P27. No other significant main effects of condition, group, or interaction of condition and group were observed in these three SEPs. As per the Wilcoxon signed-rank test, amplitude in the external condition in the healthy control group was significantly higher than that in the self-condition in N20 (*p* = 0.013), and there were no significant differences in the main effect in the schizophrenia group (Table 2).

## Discussion

In the current analysis, we examined differences in SEPs and SEFs in the primary somatosensory area, elicited by stimuli produced either by the self or an external agent in healthy controls and patients with schizophrenia. In the ANOVA analysis, we found no significant interaction between condition and group in any SEF and SEP value. This result is contrary to our expectations based on the results of previous studies of the auditory system (Ford et al., 2001a,b, 2007, 2014; Ford and Mathalon, 2004) and somatosensory system (Blakemore et al., 2000; Farrer et al., 2004) in schizophrenia.

SEP and SEF were evaluated in the current study. Both are electrical activities evoked by peripheral stimulus. They are measured by EEG and MEG, respectively. While EEG waves are distorted by electric resistance of the tissue surrounding the brain, MEG waves are not distorted but diminished by the distance from the signal origin. In addition, MEG is superior to detect tangential current but not radial current. MEG and EEG work in a complementary manner and their simultaneous measurement is very useful. In the present study, N20m in SEF and N20 in SEP showed opposite main effects of the condition. We speculated that this was due to the SEP waveform being influenced by activity and volume conduction in subcortical pathways involving the thalamus. Whereas SEF is excellent for identifying the source of cortical-derived neural activity, thus, these two modalities provided complementary information each other. On the other hand, Common main effects of group were demonstrated in both P30m in SEF and P27 in SEP. In the following discussion, we focused more on cortical-derived neural activity obtained from SEF.

A previous MEG study of healthy individuals showed that self-produced tactile stimulation attenuated somatosensory responses after 30 ms (Hesse et al., 2010). P30m was also modulated by interstimulus interval (Wikström et al., 1996), interfered stimulation (Lim et al., 2012), or motor anticipation (Wasaka et al., 2003) in the primary sensory area. In prior EEG studies with healthy subjects, SEP in the primary sensory area was shown to be suppressed when participants evoked stimulus by themselves or were given prior notice to prepare for an external stimulus (Cheron and Borenstein, 1987; Cohen and Starr, 1987; Tapia et al., 1987). These studies have shown that the perception of self-derived stimuli is suppressed in the human somatosensory system, which is confirmed by the attenuation of SEF or SEP in the primary sensory cortex.

Although many previous studies reported gating effect of P30m, the gating effect of N20/N20m has been scarcely reported. Wikström reported the effects of interstimulus interval (ISI) on SEF which demonstrated that N20m is slightly attenuated at the shortest ISI of 0.15 s. However, in other studies, gating effect of interference of motor exercise or sensory input of N20/N20m has not been found as far as we investigated. In contrast, gating effect of P30m level by various interference was repeatedly reported as attenuation of P30m in the previous studies (Wikström et al., 1996; Wasaka et al., 2003; Lim et al., 2012), those are in line with the main effect shown in this study.

On the other hand, we did not find a significant interaction of condition and group. Although no previous studies have examined corollary discharge in the primary somatosensory area in patients with schizophrenia, Thoma and colleagues showed disturbances of gating in the secondary somatosensory area but not the primary somatosensory area. This refers to a phenomenon in which response to the second stimulus in a sequence of sensory stimuli is weaker than the response to the first stimulus (Thoma et al., 2007). A recent mega-analysis of cortical brain abnormalities in patients with schizophrenia showed that the primary sensory area is one of the least impaired regions in this patient group (Van Erp et al., 2018). These findings suggest that impaired suppression of self-derived stimuli may implicate the secondary somatosensory cortex. However, because somatosensory processing regions, particularly the secondary somatosensory cortex, remain active for as long as 8 s following electrical stimulation of the periphery (Hari et al., 1993), it is difficult to confirm this hypothesis in the current paradigm.

Another possibility is that impairment of corollary discharge may also exist in the primary sensory cortex; however, we cannot detect them. The strength of P30m was not normally distributed in any of the conditions or groups (Supplementary Table 1), suggesting that the ANOVA analysis may not have yielded correct results. The *post-hoc* Wilcoxon signed-rank test revealed that the peak value of P30m was significantly higher in the external condition than in the self-condition in the healthy control group but not in the schizophrenia group. This might be suggested that, as we had initially assumed, P30m in the self-condition was suppressed compared to external condition in the healthy control only. In addition, the peak value of P30m was significantly correlated with a PANSS positive symptoms score in the schizophrenia group, only in the self-condition. If we assume that the strength of the response to self-induced stimuli is a reflection of disturbances in corollary discharge, it might be possible that the relationship between impaired corollary discharge and positive symptoms is reproduced in the current study, consistent with previous studies. In the current study, we did not clearly determine which level of somatosensory gating was impaired in subjects with schizophrenia. Further investigation with more participants may reveal the possibility that the primary sensory cortex is also affected by corollary discharge.

There are several limitations in our study. First, the sample size was small, and all samples were gathered from one hospital. This makes our finding less conclusive and less generalizable. Further studies with larger subjects from various institutions are needed to confirm our findings. Second, all patients in our study undertook neuroleptic medications at the time of testing, and thus, the effects of medication cannot be excluded. Third, patients in our study had various durations of illness (0.4–18.2 years) and varying symptom severity (the total PANSS scores ranged from 40 to 90). Further studies are needed to address the effect of pharmacological treatment, and should perhaps examine a more homogenous sample of patients (e.g., only patients with first-episode schizophrenia).

Unfortunately, we also did not obtain an index of social cognitive function in the current study, and did not examine the relationship between impaired corollary discharge in the primary sensory cortex and impaired social cognitive function. Simultaneous assessment of corollary discharge deficits, positive symptoms, and social cognitive functioning can provide useful insights into the relationship among them.

In conclusion, the present study suggests that positive symptoms such as auditory hallucinations in patients with schizophrenia may be caused by malfunction of corollary discharge in the primary sensorimotor area.

## Data availability statement

The raw data supporting the conclusions of this article will be made available by the authors, without undue reservation.

## Ethics statement

The studies involving human participants were reviewed and approved by the Hokkaido University Hospital Ethical Committee. The patients/participants provided their written informed consent to participate in this study.

## Author contributions

KY, AT, and NH contributed to the conception and design of the study, organized the database, and performed the statistical analysis. KY wrote the first draft of the manuscript. AT, NH, HS, IK, and HM checked and corrected the manuscript. All authors contributed to the article and approved the submitted version.

## Conflict of interest

The authors declare that the research was conducted in the absence of any commercial or financial relationships that could be construed as a potential conflict of interest.

## Publisher’s note

All claims expressed in this article are solely those of the authors and do not necessarily represent those of their affiliated organizations, or those of the publisher, the editors and the reviewers. Any product that may be evaluated in this article, or claim that may be made by its manufacturer, is not guaranteed or endorsed by the publisher.

## Footnotes

^1^ http://www.besa.de

^2^ https://www.r-project.org

## Abbreviations

EEG: Electroencephalography
JART: Japanese Version of National Adult Reading Test
MEG: Magnetoencephalography
PANSS: Positive and Negative Syndrome Scale
SEP: Somatosensory Evoked Potential
SEF: Somatosensory Evoked Magnetic Field
